# Infection survey, molecular, pathogenicity, and morphological characteristics of *Sarcocystis* species naturally infected water buffaloes (*Bubalus bubalis*) in Egypt

**DOI:** 10.1186/s12917-024-04408-x

**Published:** 2024-12-23

**Authors:** Lamiaa K. Elsharkawy, Safaa M. Barghash, Basma M. Abou El-Nour, Wafaa Labib, Al-Shaimaa M. Sadek

**Affiliations:** 1 Zoology and Entomology Department, Faculty of Science, Al-Azhar University (Girl’s Branch), Cairo, Egypt; 2 Animal Health Department, Desert Research Institute, Cairo, Egypt

**Keywords:** Buffaloes, Sarcocystosis, Ultrastructural, Molecular, Pathogenicity, *Sarcocystis fusiformis*, *Sarcocystis cruzi*

## Abstract

**Background:**

Sarcocystosis is a parasitic disease found worldwide, resulting from various *Sarcocystis* species. The current research was carried out in three significant economic areas in Egypt: Greater Cairo, the Nile Delta, and Upper Egypt. It aimed to investigate the occurrence of *Sarcocystis* spp. in locally bred water buffaloes *Bubalus bubalis*.

**Methods:**

To achieve this objective, 317 buffalos were slaughtered in different slaughterhouses in various regions of Egypt. Samples of heart, skeletal muscle, esophagus, and tongue were assessed using macroscopic and microscopic tests. Examination methods included direct optical observation of tissues as well as digestion and examination of the sediment obtained from the tissues. Additionally, ultrastructural features were analyzed using scanning and transmission electron microscopy. Molecular characterization was conducted through PCR, followed by nucleotide sequencing and phylogenetic analysis.

**Results:**

A total of 317 slaughtered buffaloes were examined for *Sarcocystis* during the period from September 2021 to October 2023. The prevalence of infection was recorded with 229 out of 317 (72.2%) infected with *Sarcocystis* spp. The results also showed that the prevalence of *Sarcocystis* species in females was higher than males. Based on the age of carcasses, adults (> 2 years) had a higher infection rate compared to young ones (< 2 years). Regarding seasonal variation, the highest prevalence of infection was recorded during the summer followed by spring, and then autumn, while winter had the lowest prevalence of infection. Additionally, the skeletal muscle was the most susceptible organ to sarcocystosis (87.3%) followed by the esophageal muscle (8.3%), the tongue (4.4%), and no infection in the heart muscle. The use of scanning and transmission electron microscopy allowed the identification of *S. fusiformis* and *S. cruzi* in buffaloes in Egypt. Furthermore, the *Sarcocystis* 18 S rRNA genes from skeletal tissue samples were cloned and sequenced under accession numbers OQ507387, OQ507388, and OQ507389 for *S. fusiforms*, and one OQ507391 for *S. cruzi.*

**Conclusion:**

The findings revealed a notably high prevalence of *Sarcocystis* infection (72.2%) in buffaloes from Egypt, with skeletal muscle identified as the organ most susceptible to the parasite. Two *Sarcocystis* species were detected: *S. fusiformis* and *S. cruzi.*

## Background

Sarcocystosis is a worldwide parasitic disease induced by different species of *Sarcocystis* [1]. A carnivorous (definitive host) and an herbivorous (intermediate host) are required for the cyst-forming coccidian parasite *Sarcocystis* spp. to complete its life cycle [2]. After ingesting the oocyst stage from the feces of the definitive host, intermediate hosts undergo an asexual life cycle that culminates in the development of intramuscular cysts [3]. In humans, sarcocystosis exists in two distinct forms. The first type, intestinal sarcocystosis, is a zoonotic disease resulting from the consumption of raw infected beef by *Sarcocystis fusiformis* or raw infected pork by *Sarcocystis meischeriana*. Both types can lead to gastrointestinal issues like vomiting, stomachaches, diarrhea, nausea, and dyspnea [4]. The second form, muscular sarcocystosis, arises when sporocysts are ingested, commonly through contaminated food or drink and occasionally from fresh vegetables [4]. There are over 150 different *Sarcocystis* species that are known to parasitize both domestic and wild animals. Weight loss, abortions, decreased milk output, and the mortality of infected animals are common signs of infection [5]. For a variety of Egyptian consumers, meat serves as their primary source of animal protein. Red meat consumption has become more popular in recent years across the world [6], and the demand for meat needed for human consumption will rise rapidly as the human population grows.

Ultrastructure and histology are crucial techniques for the diagnosis of muscle infection because the size, shape, and appearance of the *Sarcocystis* as well as the cyst wall are reliable criteria to distinguish this parasite from other parasites in the same phylum. Understanding this parasite’s histomorphology and how it differs from other parasites with a similar appearance is essential [7]. In recent years, polymerase chain reaction (PCR) has become a popular and reliable approach for detecting and identifying *Sarcocystis* species from a variety of hosts due to the lack of identifiable species distinctions using optical microscopy. Furthermore, the evolutionary relationships between various species may be determined thanks to molecular data, which again allows for the prediction of the likely definitive hosts of species for which the entire life cycle has not yet been determined.

Additionally, many serological diagnostic techniques are insensitive [8]. *S. fusiformis* is the dominant species of *Sarcocystis* that affects buffaloes in different regions such as Egypt, China, Iran, and India [9]. Significant genetic variation has been observed among the different *S. fusiformis* isolates worldwide [10]. *S. cruzi* was the first species described in cattle, and each *Sarcocystis* species identified in livestock demonstrates a significant specificity at the intermediate host level [11]. For instance, there is no expectation that buffalo will be susceptible to infection by species that typically infect cattle [12]. Nevertheless, instances of cross-transmission of *Sarcocystis* species between water buffalo and cattle were documented byWang [13] andBN [14], providing evidence that water buffalo can indeed be infected with *S. cruzi*. The current work is an effort to use molecular, ultrastructural, and microscopical studies to examine the prevalence of several *Sarcocystis* spp. infecting slaughtered animals in three important economic regions in Egypt and offers the most recent information on sarcocystosis in Egypt.

## Materials and methods

### Study area

**Fig. 1:**
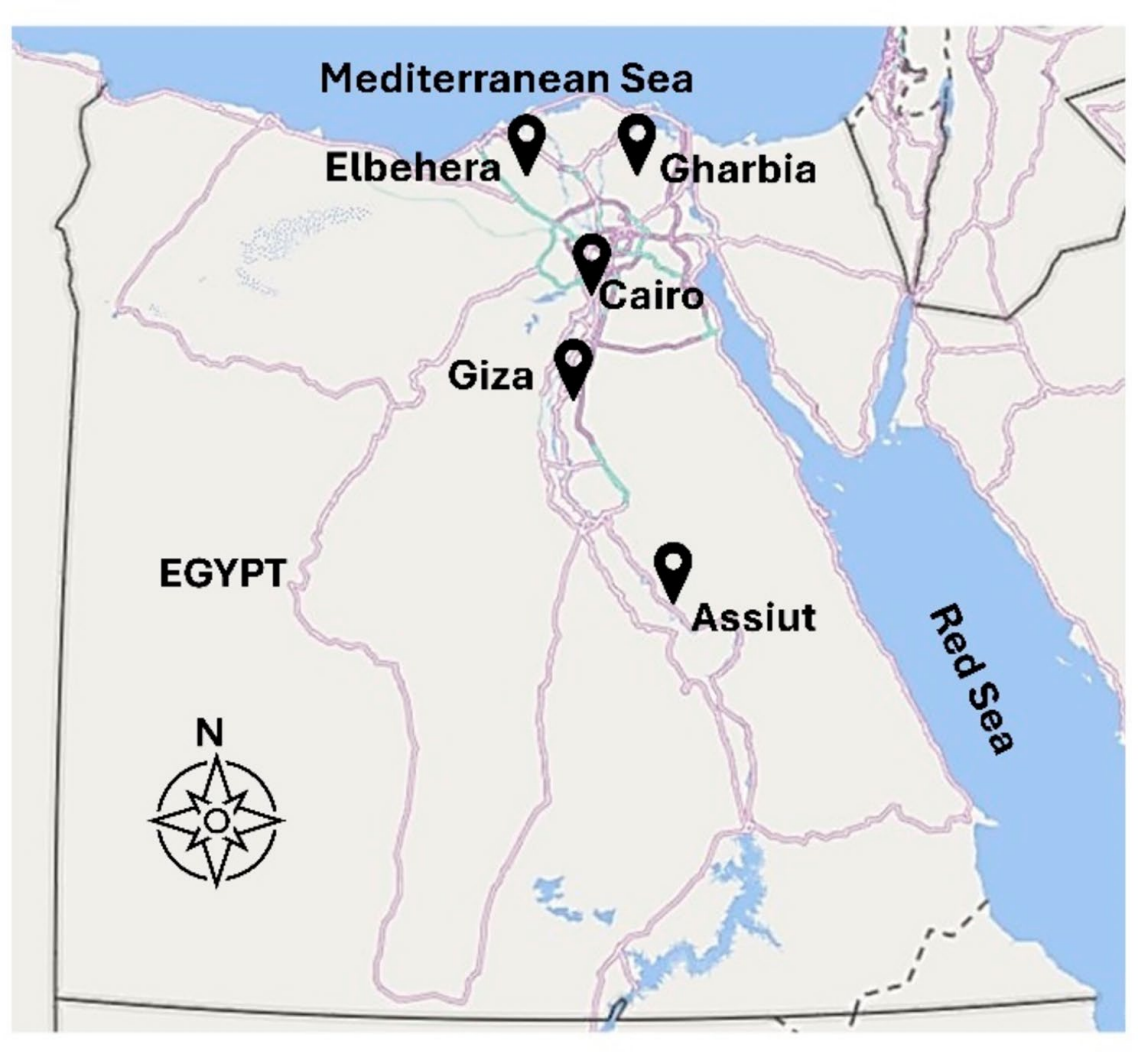
The map of Egypt shows sampling sites

Carcasses were examined at different localities in Egypt represented by three main economic regions in Egypt (Greater Cairo, Nile-Delta and Upper Egypt): Cairo [30° 1' 59.9988" N, 31° 14’ 0.0024" E] (Bassatein abattoir), Giza [29° 58’ 35.3280" N, 31° 7' 52.6872" E] (El- Moneib abattoir), Delta region: Gharbia [30° 52.5214" N, 31° 2.0106" E] and ElBehera [30° 30.8855" N, 30° 20.6131" E], and Upper Egypt: Assuit [27° 10’ 51.46" N, 31° 11’ 1.25" E] (Fig. 1).

### Animals and samples collection

The current study involved examining 317 buffaloes brought in for slaughter for human consumption in slaughterhouses across Greater Cairo (Bassatein and El- Moneib abattoirs), the Nile Delta (Gharbia and ElBehera abattoirs), and Upper Egypt (Assiut abattoir) from September 2021 to October 2023 through regular visits. To systematically record clinical symptoms in slaughtered buffaloes with sarcocystosis infection, a comprehensive examination, encompassing the observation of vital signs, behavior, and physical changes, was conducted.

### Postmortem examination

After sacrificing, the fresh muscle samples (cardiac, skeletal, lingual, and esophageal muscles) were macroscopically inspected for the existence of *Sarcocystis* cysts. Fresh tissue specimens (35–50 g) were transported from the abattoir in labeled ice bags for additional research [15]. Prior to examination, specimens were kept in the refrigerator. For microscopic examination, the squash method or compress method was used. Fresh muscle samples, measuring 2–3 mm3 in thickness, were obtained using a biopsy needle and forceps. Careful attention was given to preserving the samples’ integrity and freshness throughout the collection process to ensure accuracy in subsequent analyses. The specimens were squeezed firmly between two glass slides and inspected under a microscope at a magnification of 10x [16].

### Scanning electron microscopy (SEM)

Fresh muscle samples containing cysts (50 g each) were gathered from the infected animals. These samples were carefully placed in clean plastic bags and transported in a clean ice box, and then cysts were delicately extracted from fresh muscular fibers using fine forceps and needles for preparing the samples for examination under examination under scanning electron microscopy (SEM) and transmission electron microscopy (TEM). For SEM analysis, cysts were fixed in 70% formaldehyde-glutaraldehyde mixture. Subsequently, dehydration was carried out using ethanol at varying concentrations, followed by immersion in hexamethyldisilazane (HMDS) for 5 min and mounting on stubs with double sticky tabs. Gold coating was applied, and the samples were examined using a JEOL GM 5200 microscope [17]. This entire process took place at the Applied Center for Entomonematodes (ACE), Faculty of Agriculture, Cairo University, Giza, Egypt.

### Transmission electron microscopy (TEM)

Tiny, undamaged cysts with minimal adjacent tissue were separated and preserved in a solution of 3% glutaraldehyde in 0.1% sodium cacodylate (pH 7.4). After washing in the same buffer, they underwent post-fixation with 2% OsO4 in the same buffer. The tissue fragments were dehydrated through a series of ethanol gradients and then embedded in araldite. Ultra-thin sections were stained with uranyl acetate and lead citrate before examination with a Philips (208) electron microscope operating at 80–100 kV, as detailed by [18]. The technique was conducted at the regional center of fungi and its application, Al-Azhar University, Cairo, Egypt.

### Histopathological studies

According to [19], positive muscular specimens were preserved in 10% formalin, then cut into sections and stained with Ehrlich’s Hematoxylin and Eosin.

### DNA extraction and PCR amplification

Genomic DNA was extracted from positive samples during macroscopic and microscopic examinations of different muscle tissues of the heart, skeletal, lingual, and esophageal, according to QIAamp DNA mini kit instructions, catalog No. 51,304 (QIAGEN Technical Services, USA). PCR amplification was performed according to the Emerald Amp GT PCR master mix Takara kit (Takara Bio Inc., San Jose, California, USA) Code No. RR310Akit. The 18 S rRNA molecule was species-specific, targeting the Sarc cattle gene by amplifying a 600-bp fragment used as a molecular marker in phylogenetic analysis. Their sequences were Sarc-F: GCA CTT GAT GAA TTC TGG CA and Sarc-R: CAC CAC CCA TAG AAT CAA G, according to [20]. Thermal cycling started at 94 °C for 5 min, followed by 40 cycles of 94 °C for 2 min, 55 °C for 1 min, and 72 °C for 90 s, followed by a final elongation step at 72 °C for 5 min. PCR product samples and negative and positive controls were loaded onto a 1.5% agarose gel., stained with ethidium bromide, and the gels were photographed with UV transillumination. After that, four sharp PCR-positive samples were chosen from three different tissues for gene sequencing. The amplified PCR products were purified using a Takara Bio Inc. (San Jose, California, USA) kit and cloned into a pMD19-T vector (Takara Bio Inc., San Jose, California, USA) kit.

### Gene sequencing and phylogenetic analysis

Generated fragments were subjected to a 2-way sequence analysis using the ABI 3130 automated DNA sequencer (Applied Biosystems, Thermo Fisher Scientific, Foster City, CA, USA) using the same forward and reverse primers for PCR. Sequences were submitted to GenBank and blasted with various *Sarcocystis* spp. They sorted using the Clustal W algorithm. Sequence similarities compared to preserved sequences of closely related species. The phylogenetic tree was reconstructed by neighbor-joining, and similarity between isolates was determined using the maximum likelihood test in MEGA6 software [21].

### Statistical analysis

Chi-square tests were used to evaluate the relationship between the variables (gender, age, place of origin, seasonal changes, and organ susceptibility) and the presence of *Sarcocystis* spp.

## Results

### Prevalence of sarcocystosis

**Fig. 2:**
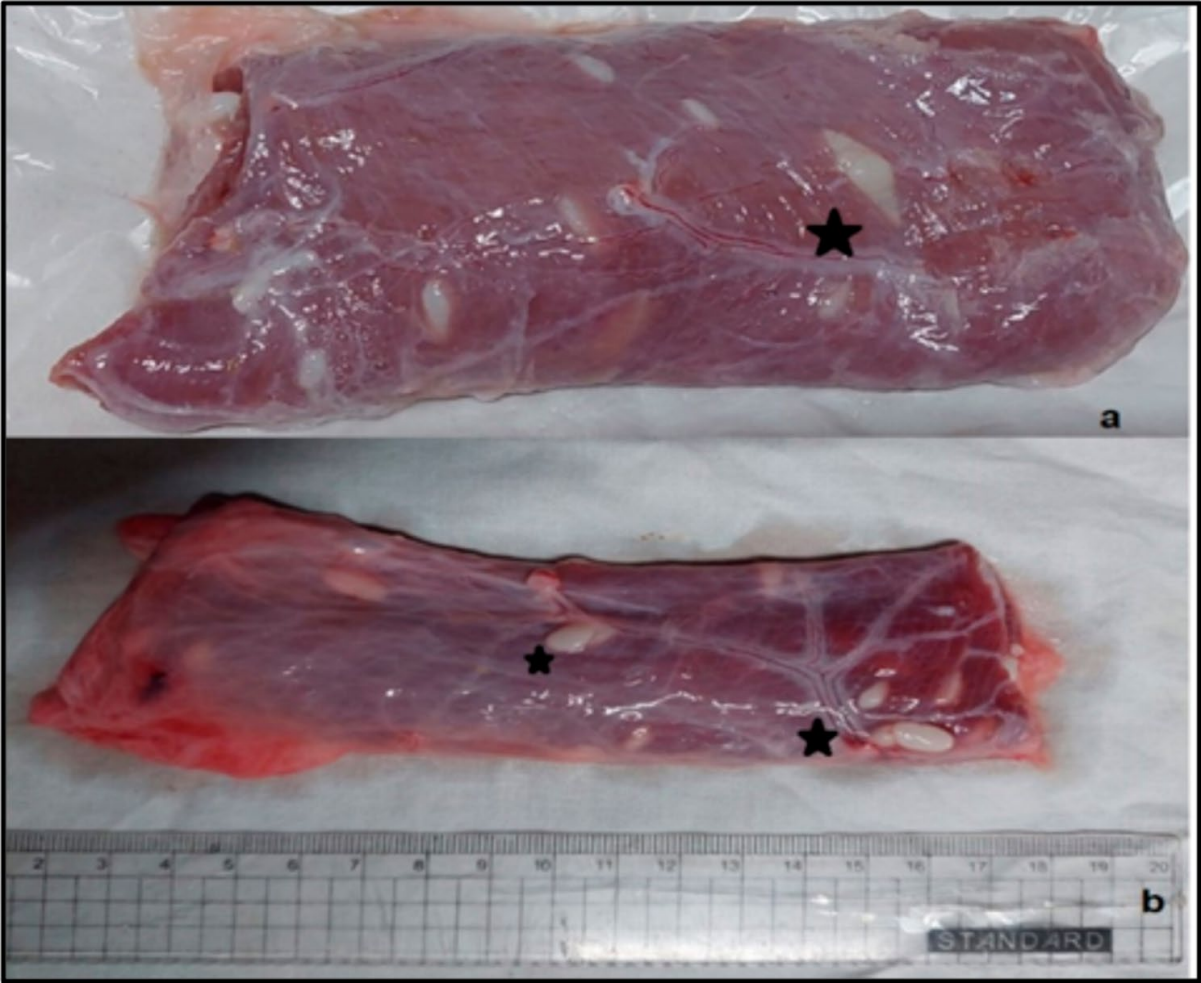
Photomicrograph of esophageal muscle showing cysts of *Sarcocystis sp*. embedded in the muscle fibers of the host, (**a**) showing an elongated fusiform cyst of *Sarcocystis fusiformis* (black star). (**b**) showing the rounded ends of the cyst of *Sarcocystis cruzi* (black stars)

Out of the 317 buffaloes examined for natural infection with *Sarcocystis*, 229 tested positive (72.2%) Slaughtered buffaloes suffering from sarcocystosis infection exhibited various symptoms, including fever, loss of appetite, reduced milk production, diarrhea, hair loss on the tail, muscle spasms, increased sensitivity and restlessness, general weakness, and extreme fatigue. *Sarcocystis sp*. cysts were clear and easily distinguished by the naked eye in 214 specimens (93.4%), while only 15 specimens (6.5%) required microscopic examination using the squash method. The slaughterhouses in the Greater Cairo region recorded the highest frequency of positive cases (139/229) (60.6%), followed by West Delta slaughterhouses (66/229) (28.8%) while the lowest prevalence of infection was recorded in Upper Egypt slaughterhouse (24/ 229) (10.5%). The prevalence of *Sarcocystis* species in females and males was 84.7% and 57.8%, respectively. The results showed that there were significant differences in the infection rate between males and females, as the chi-square value reached 5.098. Based on the age of carcasses, adults (> 2 years) had a higher infection rate compared to young ones (< 2 years), with rates of 78.5% and 59.8%, respectively. Regarding seasonal variation, the highest prevalence of infection was recorded during summer (50.9%), followed by spring (21.6%), and then autumn (14.3%), while winter had the lowest prevalence of infection (12.5%). The results showed that there were significant differences in the infection rate between the seasons, as the chi-square value reached 38.00, which is significant at a significant level of 0.01. Additionally, the result showed that the skeletal muscle was found to be the most susceptible organ to sarcocystosis (87.3%), followed by the esophageal muscle (8.3%), and the tongue (4.4%). No infection was observed in the cardiac muscle. The results showed that there were significant differences in the infection rate between the affected organs, as the Chi-square value reached 132.788, which is significant at a significance level of 0.01 (Fig. 2 & Table 1).

**Table 1:** Prevalence of sarcocystosis in buffaloes according to gender, age, seasonal variations, and infected organ

Variable		Examined	Infected (%)	Chi-Square	*P*- value
Sex	Female	170	144 (84.7%)	5.098	0.024
	Male	147	85 (57.8%)		
Age	adults (> 2 years)	210	165 (78.5%)	2.597	0.107
	young (< 2 years)	107	64 (59.8%)		
Season	Summer	106	54 (50.9%)	38.00	0.000
	Spring	60	13 (21.6%)		
	Autumn	63	9 (14.3%)		
	Winter	88	11 (12.5%)		
Infected organ	Skeletal muscle	229	200 (87.3%)	132.788	0.000
	Esophagus	229	19 (8.3%)		
	Tongue	229	10 (4.4%)		
	Cardiac muscle	229	0 (0.0%)		

### Histopathological results

**Fig. 3:**
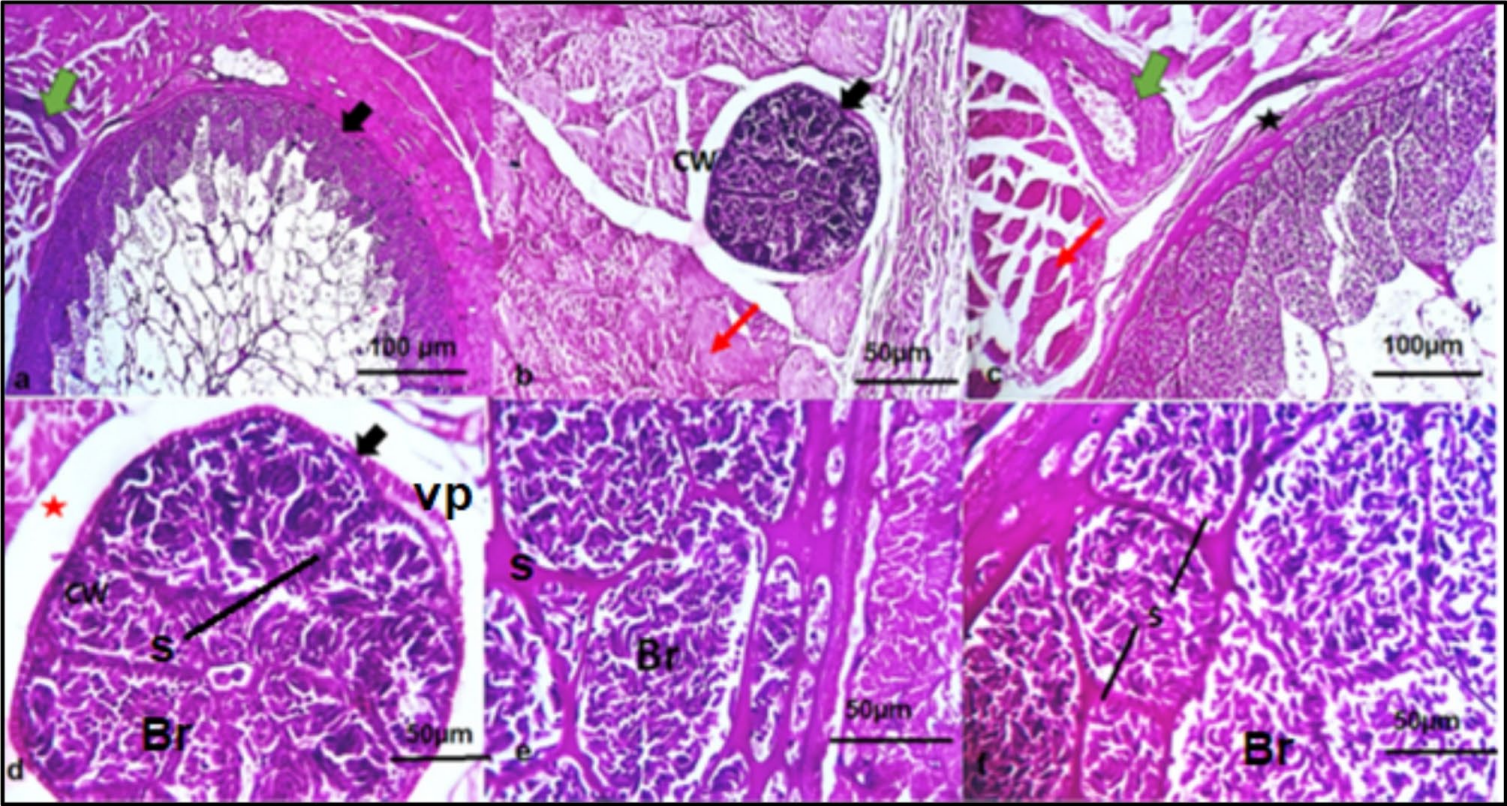
A photomicrograph from the skeletal muscles of the infected cattle shows heavy sarcocystosis infection with the presence of both macro and microcysts (black arrows) (**a**, **b** & **c**). The cyst is enclosed by a cyst wall (CW) (**b**) that is underlined by a layer of ground substance. This ground substance extends into the inner side of the cyst, forming septa (S) containing bradyzoites (Br) (**d**, **e**, & **f**). A thin fibroblastic tissue capsule can be seen surrounding large cystic lesions (black aster) (**c**). Mild vascular dilatation (green arrows) (**a** & **c**) edema (red aster) (**d**) and hyaline degeneration (red arrows) (**b& c**) are seen. Note that finger-like villar protrusions (vp) are clearly visible on the striated cyst wall

Sarcocystosis manifests as a condition characterized by infestation with the dormant stage (bradyzoites), leading to cystic development in multiple areas within and between the muscles. These cysts can vary in size from a few micrometers to centimeters and occasionally exhibit multilocular cystic forms. The histopathological results are all presented in Fig. 3.

### Ultrastructural characteristics of *Sarcocystis* spp. in the detected skeletal tissues

**Fig. 4:**
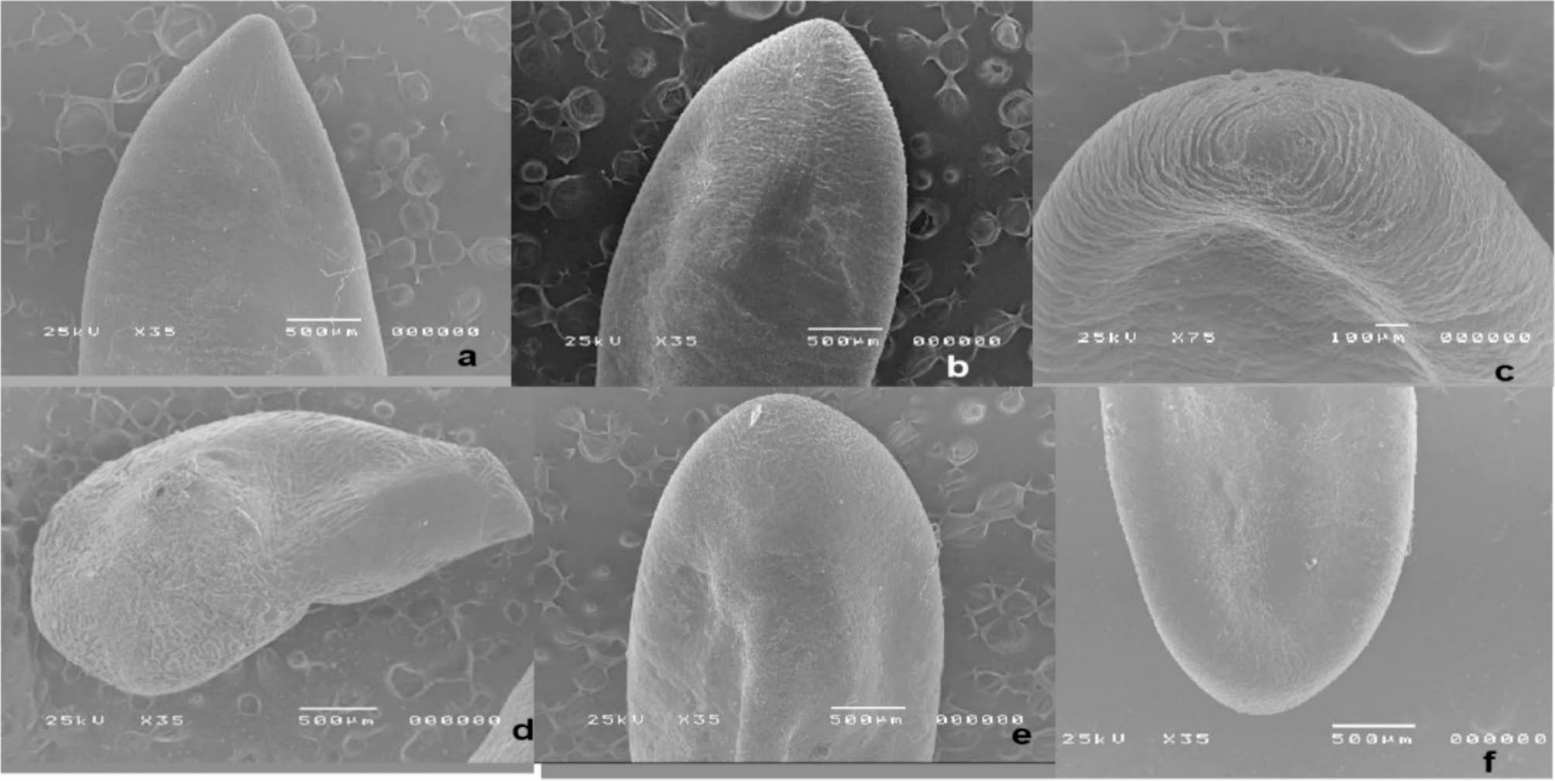
Scanning electron microscope (SEM) photos, **a & b**: showing fusiform cyst of *Sarcocystis fusiformis*, **c-f**: showing the cyst of *Sarcocystis cruzi.* **c**: showing radially striated protrusions folded over the surface. **d**: showing both convex and concave sides of the cyst. **e & f**: showing both rounded ends of the cyst

**Fig. 5:**
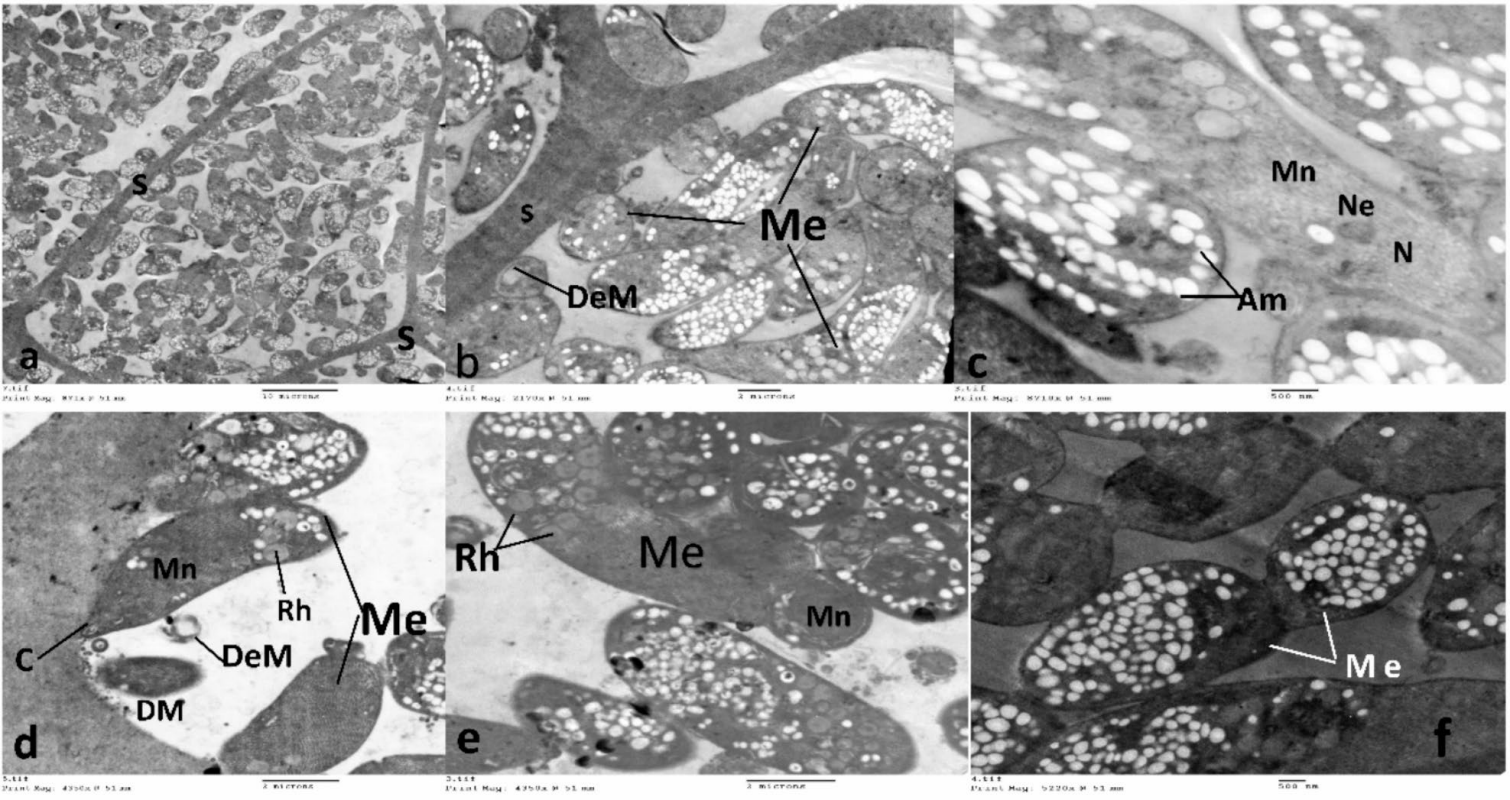
TEM of *Sarcosystis* sp. showing (**a**) the cyst is divided into many chambers separated from each other by the septa (S). (**b**) higher magnification of chambers with large numbers of merozoites (Me) and degenerated merozoites (DeM). (**c**) higher magnification of merozoites with micronemes (Mn), clear nucleus (N) with a nucleolus (Ne) in addition to amylopectin granules (Am). (**d**) clear banana-shaped merozoites (Me) with micronemes (Mn), rhoptries (Rh), and conoid (C), also figure showed degenerated (DeM) and developed (DM) merozoites. (**e**) well-developed merozoite (Me) with micronemes (Mn), and rhoptries (Rh). (**f**) the division of merozoites (Me)

The skeletal tissue has many cysts that are visible to the naked eye. The scanning electron microscope revealed that the cyst of *S. fusiformis* had a spindle shape, measuring 149–224 μm length × 33–133 μm in width, with the outer cell wall covered with fine hair-like protrusions (Fig. 4a &b). On the other hand, the cyst of *S. cruzi* exhibited rounded anterior and posterior ends, with an outer cell wall showing radially striated protrusions folded over the surface. The dimensions of the cyst were 35–110 μm in width and 66–300 μm in length. Additionally, one side of the cyst was convex, while the other side was partially concave in the middle (Fig. 4c, d, and e). The transmission electron microscope revealed the presence of cysts typically found in the host’s muscle tissues, and their aggregation forms. *Sarcocystis*, which contain numerous merozoites in chambers separated by internal septa (Fig. 5).

### Molecular characterization of *Sarcocystis* species

**Fig. 6:**
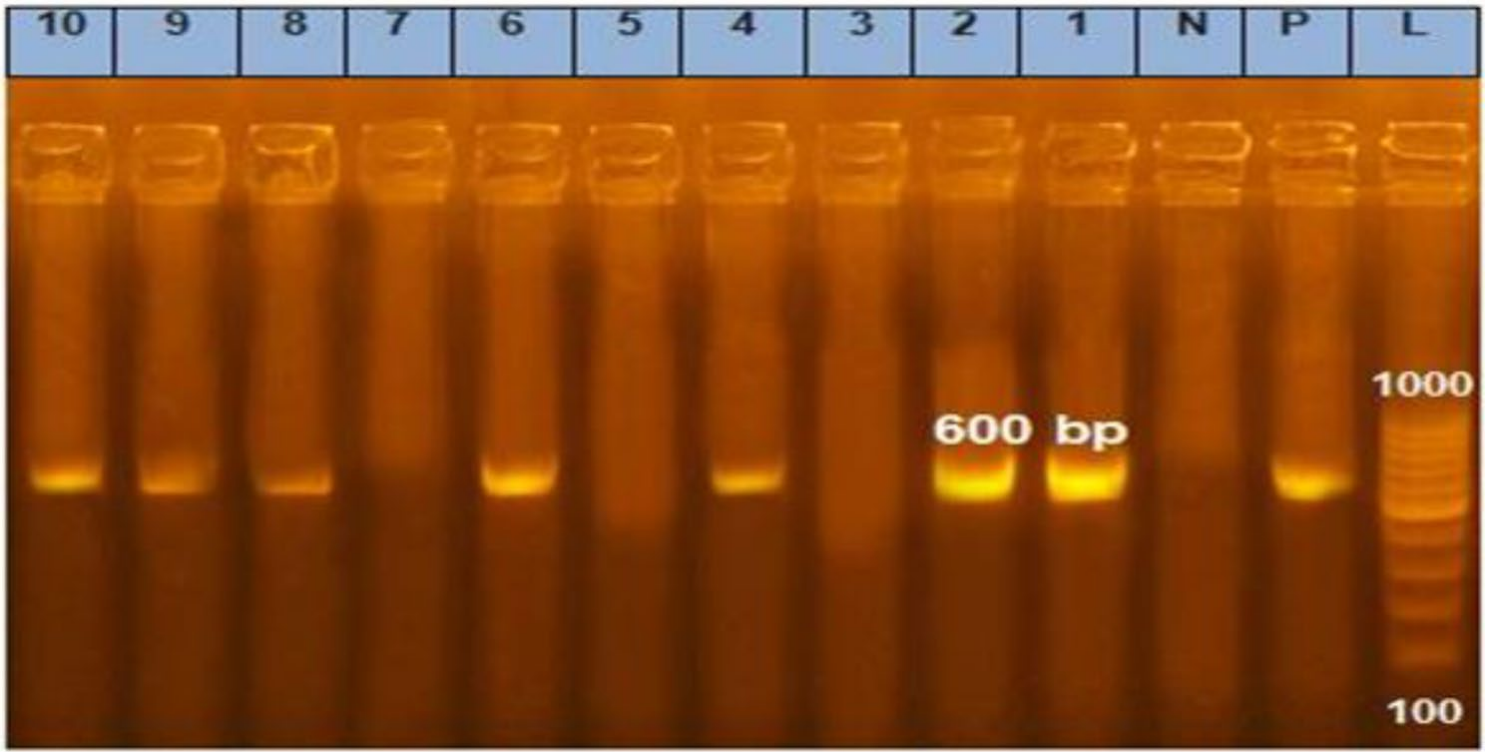
PCR-based assays targeted the 18S rDNA gene for *Sarcocystis*. L, L lanes: 100 bp DNA ladder. Lanes 1, 2, 4, 6, 8, 9, and 10 correspond to positive results for *Sarcocystis*. Lanes P and N represent positive and negative controls, respectively.

In the present study, *Sarcocystis* produced distinct bands at 600 bp in 70% of the DNA-extracted samples that were recovered from water buffaloes slaughtered in different governorates, and it was absent in 30% of the samples as shown in Fig. 5. When the newly isolated nucleotide sequences from this investigation were compared using BLAST with existing sequences in GenBank, DNA from two separate *Sarcocystis* spp. were discovered. Of the four *Sarcocystis* identified, three belonged to *S. fusiformis* and the fourth to *S. cruzi* (Table 2). It revealed genetic sequences of *S. fusiform* and *S. cruzi* sequences related to other species in the genus *Sarcocystis.* Their amplicons yielded by 18 S rRNA had 600 base pairs (bp) (Fig. 6). The 18S rRNA gene analysis of the detected *Sarcocystis* revealed 100% identity and similarity with the corresponding sequences. The following accession numbers have been assigned to the nucleotide sequences from this investigation in GenBank: OQ507387, OQ507388, and OQ507389 for *S. fusiforms*, and one OQ507391 for *S. cruzi*.

**Table 2:** The GenBank database of identified *Sarcocystis* species isolated from Water buffaloes in Egypt

*n*	ID	organ	Date	Parasite
1	*Sarc1-Bu-AZ-DRC*	Muscle	1-4-2022	OQ507387 (*S. fusiforms*)
2	*Sarc2-Bu-AZ-DRC*	Muscle	5-6-2022	OQ507388 (*S. fusiforms*)
3	*Sarc3-Bu-AZ-DRC*	Muscle	8-9-2022	OQ507389. (*S. fusiforms*)
4	*Sarc4-Bu-AZ-DRC*	Muscle	7-10-2022	OQ507391 (*S. cruzi*)

**Fig. 7:**
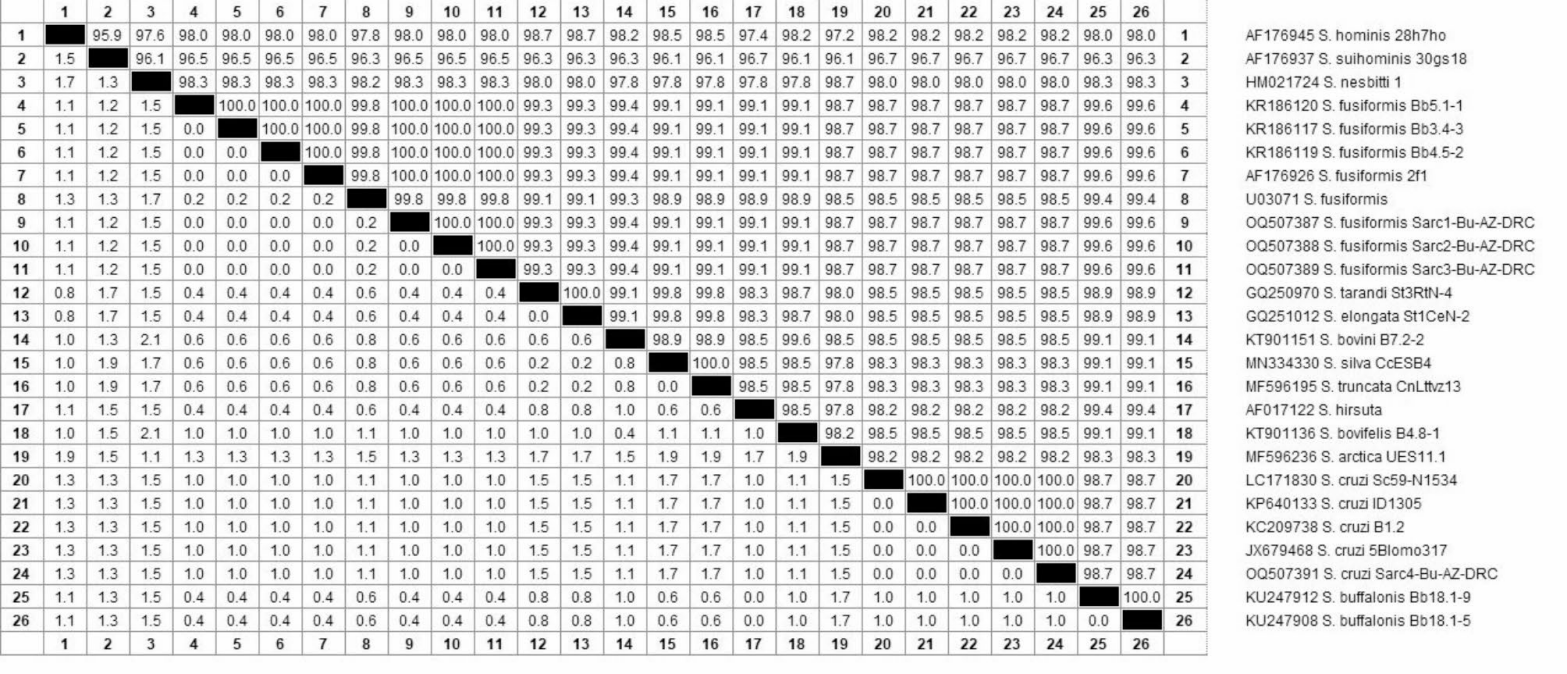
The percentage of identity for our Sarcocystis fusiformis and Sarcocystis cruzi isolates based on the 18S rRNA gene. Their accession numbers are followed by (AZ-DRC)

**Fig. 8:**
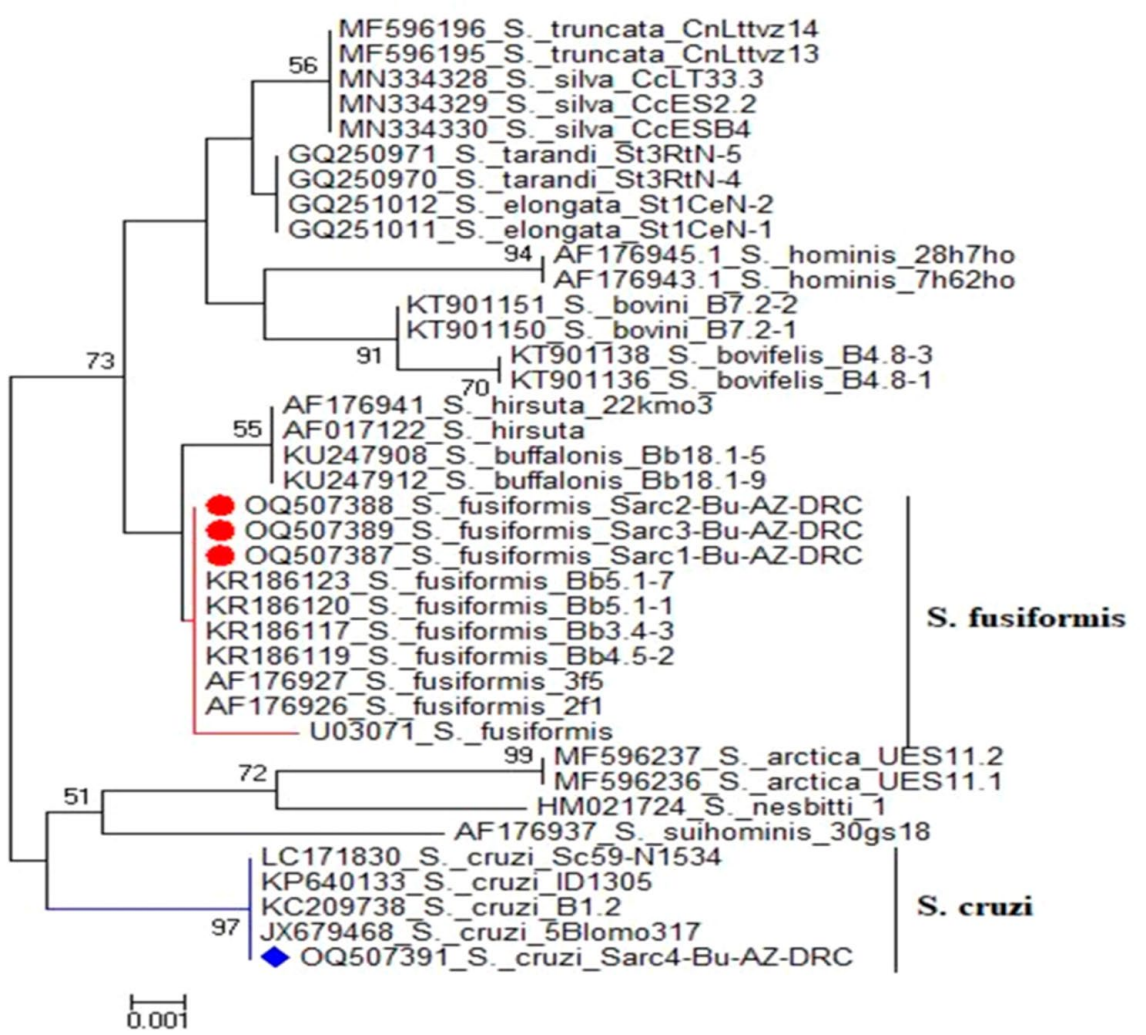
The phylogenetic relationship of the 18S rDNA gene for submitted isolates of Sarcocystis fusiformis and Sarcocystis cruzi

With regard to the phylogenetic analysis and sequencing of *Sarcocystis*, it was discovered that a 527 nucleotide-long partial DNA sequence of 18 S rRNA was detected, which was compatible with the anticipated amplified size. The sequence identity between the three *S. fusiform* isolates was 100%, but the identity between the isolates of *S. cruzi* and *S. fusiform* under study was 98.7% when compared to that preserved in the GenBank database as shown in Fig. 7. At the 18 S rRNA gene, the nucleotide sequences of *S. fusiform* and *S. cruzi* could be separated. However, four nucleotides were replaced by T, T, and Cat positions 316, 318, 320, and 321. After comparing the recently produced 18 S rRNA gene sequences of *S. fusiform* from water buffaloes with comparable sequences that were preserved in GenBank, it showed that *S. fusiform* is a species that differs from *S. cruzi* and is closely linked to *S. hirsuta*. The phylogenetic tree of the 18 S rRNA sequence showed that the 18 S rRNA sequences of *S. fusiformis* and *S. cruzi* split into two clusters, as shown in Fig. 8.

*S. fusiformis* from other countries, *S. buffalonis*, and *S. hirsute* were the closest similarities between the several *Sarcocystis* spp. that were previously investigated in GenBank with some genetic changes and *S. fusiformis* under research. They were grouped in the same cluster as *S. truncata*, *S. tarandi*, *S. elongata*, *S. silva*, *S. bovini*, *S. hominis*, and *S. bovifelis*, along with other *Sarcocystis* species. The species *S. arctica*, *S. nesbitti*, and *S. suihominis* were similarly related to *S. cruzi*. For *S. fusiformis* and *S. cruzi*, the genetic distance divergence between those isolates varied from 0.0 to 1.7 and from 0.4 to 1.3, respectively. The isolates of *S. fusiformis* under study clustered with some Egyptian isolates and others previously identified and maintained in GenBank with accession numbers KR186123, KR186117, and KR186119 (Egypt), U03071 (Sweden), AF176927, AF176926 (China), whereas *S. cruzi* under study was clustered with accession numbers: LC171830 (Japan), KP6401133 (USA), and KC209738, JX679468 (Argentina), as shown in Table 3.

**Table 3:** GenBank database of detected *Sarcocystis* species

Parasite	Accession Number	Host	Country	Reference
***S. fusiformis***	OQ507387, OQ507388, OQ507389	*Bubalus bubalis*	Egypt	The present study
	KR186123, KR186117, KR186119	*Bubalus bubalis*	Egypt	[10]
	U03071	*Felis catus*	Sweden	[22]
	AF176927, AF176926	*Bubalus bubalis*	China	[23]
***S. cruzi***	OQ507391	*Bubalus bubalis*	Egypt	The present study
	LC171830	*Bos taurus*	Japan	[24]
	KP6401133	*Bison athabascae*	USA	[25]
	KC209738, JX679468	*Bos taurus*	Argentina	[26, 27]
	MF360017	*Bos taurus*	Iran	[28]
	OL305830	*Bos taurus*	Egypt	[5]
	MN832695	*Bos taurus*	Türkiye	[29]
	MT792460	*Bos taurus*	Lithuania	[1]

## Discussion

*Sarcocystis* species are common protozoan parasites found in domestic animals. Certain *Sarcocystis* species lead to economic losses due to both clinical and subclinical diseases, as well as the condemnation and downgrading of carcasses [30]. *Sarcocystis* species typically undergo two host cycles: one in the intermediate host and another in the definitive host. Multiple *Sarcocystis* species can infect a single host [31]. The present study observed a notable prevalence of *Sarcocystis* infection in water buffaloes slaughtered at various Egyptian slaughterhouses. The elevated occurrence of *Sarcocystis* infection in water buffaloes is likely a consequence of their close interactions with dogs, cats, and other wildlife, which act as definitive hosts for the parasite [4]. Contributing factors to this heightened prevalence encompass the extensive shedding of infectious sporocysts, the prolonged resistance of oocysts or sporocysts in the external environment, the involvement of invertebrates as intermediary hosts in the infection, and the absence or limited immunity to the re-shedding of sporocysts after consumption of infected meat [32]. All these factors collectively contribute to the high prevalence of sarcocystosis. We hypothesize that the elevated occurrence of macroscopic *Sarcocystis* spp. in our data, which excludes any microscopic forms, may be attributed to frequent contact between infected cats and dogs (definitive hosts) and water buffaloes [33].

The overall *Sarcocystis* macrocyst prevalence among the carcasses of buffaloes in this investigation was consistent with that found in Egypt by Ghaffar et al. [34] Abu-Elwafa et al. [35]; El-Bahy et al. [36]; and El Shanawany et al. [37], they found that the *S. fusiformis* infection rate in water buffaloes was 100%, 58.72%, 85.96%, and 74%, respectively. On the other hand, our results were higher than those reported in investigations conducted by Khalifa et al. [38]; El-Dakhly et al. [33]; and Ahmed et al. [39], who recorded infection rates of 28%, 6.9 and 8.33% respectively. Other countries with comparable climates have reported higher infection rates; for example, 87% in India [40] and 82.9% in Iraq [41].

The current study found a slightly higher prevalence of *Sarcocystis sp*. infection in female water buffaloes when compared to male. These results align with the observations of Ahmed et al. [39]; Ibrahim et al. [42]; and El Shanawany et al. [37], who also noted a higher proportion of infected females compared to males. This contrasts with earlier research by Oryan et al. [30]; Ras [43]; and Ghorbanpoor et al. [44], which indicated that the proportion of infected females was lower than that of infected males. One possible explanation for the higher infection rates in females could be the added stress factors experienced by females, such as pregnancy and lactation, which may weaken the immune system [42].

In relation to the influence of age on the prevalence of *Sarcocystis sp*., our study revealed that buffaloes older than two years had a higher likelihood of infection compared to younger ones. Moreover, this investigation established a statistically significant correlation between older age and a higher *Sarcocystis* infection rate in water buffaloes. These findings align with prior research by Huong [15]; Ibrahim et al.; [42] and Ras [43] all of which demonstrated a higher prevalence of *sarcocystosis* in older animals. El-Bahy et al. [36] also noted a higher infection rate in animals over the age of five (92.28%) compared to those aged three to five (29.32%). This association can be partially explained by the likelihood that older animals have been exposed to sporocyst infections for longer periods, and macroscopic cysts take longer to manifest in muscles [39].

Concerning the seasonal occurrence of *Sarcocystis* infection in water buffaloes, the study revealed significant variations throughout the year. The prevalence of *Sarcocystis* infections tended to be higher during the warmer seasons, particularly in summer and spring. This trend can likely be attributed to the extended grazing periods observed in the summer season, as noted by Huong [15]. These results align with a previous study, which found that the highest prevalence among buffaloes occurred in winter and autumn, while the highest prevalence was in summer and spring [36]. Our results are different from those of [45], who found that the colder seasons had the highest infection prevalence.

According to the site of infection, the highest prevalence was recorded in the skeletal muscle (87.3%), followed by the esophageal muscle (8.3%), and the tongue (4.4%). No infection was observed in the cardiac muscle. These results align with the findings of Daryani et al. [46] and Hornok et al. [47] who also noted that the skeletal and abdominal muscles were the most susceptible to *Sarcocystis* infection. However, in contrast, *Sarcocystis* was predominant in the esophageal muscle according to Nourani et al. [48]; and Ahmed et al. [39].

Histological analysis showed hyaline degeneration, moderate vascular dilatation, bleeding, and edema. Our findings correlate with those of Oryan et al. [49] who observed that tissues exhibited reactions to *Sarcocystis*, which could be seen as leukocytic infiltrations and hemorrhagic foci. According to Valinezhad et al. [50], bleeding was observed as an inflammatory response in the heart muscles. Furthermore, severe blood vessel congestion and degenerative alterations, such as cloudy swelling, the formation of hyaline tissue, and eosinophil infiltration, were noted by JyothiSree et al. [51]. Toxins released from the cysts may be the cause of any inflammatory responses and hyalinization of the muscle [52].

In our investigation, ultrastructural analyses revealed the spindle-shaped cyst of *S. fusiformis*, measuring 149–224 μm in length and 33–133 μm in width. Correspondingly, Ardalan [53] documented analogous findings in *S. fusiformis-* infected buffaloes in Iraq. On the other hand, the cyst of *S. cruzi* displayed rounded anterior and posterior ends and the cyst’s dimensions were recorded at 64.1 μm in width and 170.9 μm in length, aligning with earlier research outcomes reported by [54].

The identification of *Sarcocystis* species in cattle has frequently relied on 18 S rDNA sequences, which can correspondingly be applied to reconstruct the evolutionary relationships among *Sarcocystis* species [29]. Because of its highly conserved character, this gene’s discriminatory capacity has been demonstrated to be inappropriate for differentiating between closely related lineages of *Sarcocystis* in ruminants [55]. On the contrary, the current work has shown that sequence analysis is more effective and sensitive in identifying *Sarcocystis* in water buffalo muscle samples. Earlier studies on both domestic and wild pigs have found similar results [56–58].

The present results of sarcocystosis also agree with the results of a recent study in Egypt by Barghash [59], who used the same primer targeting the Sarc-cattle gene amplified at 600 bp to molecular screen 353 blood samples from ruminants for sarcocystosis and the results showed that goats and sheep have no *Sarcocystis* infection, whereas the infection was 38.94% in cattle with its risky pathogenesis. Hu et al. [55] have shown that some species show low intraspecific sequence variation in a specific region, so using a single marker alone might not be sufficient and could not establish the evolutionary link between *Sarcocystis* spp. In the present study, using the 18 S rDNA gene alone could detect the phylogenetic relationship between submitted isolates of *S. fusiformis* and *S. cruzi* among others and was successful in differentiating and comparing the present isolates with 18 S rDNAs preserved in the GenBank database.

The newly sequenced four isolates from the two species of *Sarcocystis* showed high intraspecific similarities, with 100% of the *S. fusiformis* genes clustered with some Egyptian isolates and others previously identified in *Bubalus bubalis* and *Felis catus* and deposited in GenBank from Egypt [10], Sweden [22], and China [23]. Whereas the *S. cruzi* genes share similarities with others identified from *Bubalus bubalis*, *Bos Taurus*, and *Bison athabascae* and recorded in Japan [24], the USA [25], Argentina [26, 27], and Iran [28]. Based on their 18 S rDNA sequences, the present study established the evolutionary link between genes of *S. fusiformis* and *S. cruzi*, as other *Sarcocystis* spp. According to current research, *S. cruzi* and *S. fusiformis* are grouped into 2 separate clusters. Those species shared a common ancestor, and two variants of the ancient species gradually split off and adapted to live in water buffaloes, according to Gazzonis et al. [60].

## Conclusion

This comprehensive study investigated the prevalence and characteristics of *Sarcocystis* spp. in locally bred water buffaloes across significant economic regions in Egypt. The findings revealed a notable prevalence of *Sarcocystis* infection, with 72.2% of the examined buffaloes testing positive. The study elucidated variations in infection rates concerning gender, age, and seasonal factors, shedding light on higher prevalence among adult buffaloes, particularly during the summer months. Skeletal muscles emerged as the most susceptible organ to sarcocystosis. Moreover, the use of electron microscopy facilitated the identification of specific *Sarcocystis* species, including *S. fusiformis* and *S. cruzi*. The cloning and sequencing of *Sarcocystis* 18 S rRNA genes provided valuable genetic data. In summary, the study’s comprehensive insights into the epidemiology and characteristics of *sarcocystosis* in Egyptian water buffaloes contribute significantly to the understanding of this parasitic infection.

## Data Availability

The datasets supporting the conclusions of this article are included within the article (and its additional files).
